# Cycling-related orthopaedic fractures admitted to the Major Trauma Centre in the cycling capital of the UK

**DOI:** 10.1007/s00402-021-04097-3

**Published:** 2021-08-03

**Authors:** Omar Jamil, Sofyan Al Shdefat, Zaki Arshad, Azeem Thahir, Fahim Anwar, Benjamin M. Davies, Daud T. S. Chou

**Affiliations:** 1 University of Cambridge School of Clinical Medicine, Cambridge Biomedical Campus, Box 111, Cambridge, CB2 0SP UK; 2 Cambridge Orthopaedic Trauma Unit Addenbrooke’s Hospital Major Trauma Centre, Cambridge University Hospitals NHS Foundation Trust, Cambridge, UK; 3 Department of Neurosciences, Addenbrooke’s Hospital, Cambridge, UK; 4 Department of Surgery, University of Cambridge, Cambridge, UK

**Keywords:** Cycling, Fractures, Trauma, Road traffic accident, Injury pattern, Orthopaedic injuries, Fractures, Road safety

## Abstract

**Introduction:**

The popularity of cycling in the United Kingdom is increasing, with a further rise likely due to recent government cycling promotion schemes. This study aims to characterise fractures sustained due to cycling-related collisions in patients presenting to a Major Trauma Centre, in the region with the highest cycling rates in the United Kingdom.

**Methods:**

A retrospective analysis of cycling injuries presenting to our centre between January 2012 and December 2020 was performed using a prospectively collected electronic database. Comparison of fracture characteristics was made according to patient age and mechanism of injury (collision with a motorised vehicle versus collision with a non-motorised object.).

**Results:**

Of the 737 patients who suffered a cycling-related injury, 292 (39.6%) suffered at least 1 fracture to the appendicular skeleton. Overall, fractures were most commonly seen in those over 50 years of age. Upper limb fractures were more common than lower limb fractures. Fractures sustained during motorised injuries were more likely to require surgical intervention than those sustained during non-motorised collisions.

**Conclusion:**

This study provides valuable information regarding the nature, epidemiology and treatment of fractures sustained following cycling-related accidents, adding to the paucity of similar literature in the field. Given the likely increase in future cycling uptake, our results are important to clinicians treating patients with cycling-related injuries and policymakers designing safety interventions.

**Supplementary Information:**

The online version contains supplementary material available at 10.1007/s00402-021-04097-3.

## Introduction

The use of pedal cycles as a means of transport in the United Kingdom (UK) has rapidly increased, with a 32% increase in the number of miles cycled between 1998 and 2018 [1, 2]. During the recent national lockdowns due to the COVID-19 pandemic, Department for Transport statistics show an increase in cyclist traffic of up to 384% on certain days [3].

The UK government has also recently launched a £2 billion initiative to promote bicycle use through schemes such as £50 cycle repair vouchers, national cycling and walking commissioner, cycle to work scheme, pop-up cycle lanes, safer junctions, protected cycle-only corridors, and potentially a ‘cycle tube’ network above London’s underground [4, 5]. This campaign aims to tackle high rates of obesity, cut carbon emissions, and reduce virus transmission on overcrowded public transport during the COVID-19 pandemic [4]. Transport Secretary Grant Shapps states, ‘as we look to the future, we must build a better country with greener travel habits, cleaner air, and healthier communities’ [4].

These initiatives suggest that the number of cyclists using UK roads is likely to increase in the next few years. During 2019, cyclists accounted for 8.1% of all road accidents in the UK [6]. When considering miles travelled, cyclists had an accident rate of 5,051 accidents per billion miles travelled, the second-highest accident rate of all vehicles, behind motorcycles [6]. With this potential further increase on the horizon, it is important to consider cyclist safety and implement effective interventions. Furthermore, it is crucial to make clinicians aware of injury patterns and characteristics of cycling injuries, which may have implications for patient management. There is currently a paucity of literature describing the nature of cycling injuries from an orthopaedic perspective. One study characterising orthopaedic injuries, specifically in electric bicycle (e-bike) users has been published previously [7], whilst another describes fractures sustained by cyclists following tram system related injuries [8]. However, despite an increase in the uptake of e-bikes, the use of e-bikes is still much lower than that of traditional cycles, with recent figures suggesting over 60,000 e-bikes were sold in the country in 2018, compared to 3 million traditional cycles [9]. It is, therefore, possible that such studies may not be representative of cyclists as a whole.

This study aims to characterise orthopaedic fractures (affecting the appendicular skeleton and sacrum) sustained by all patients admitted with cycling-related injuries in 9 years, to a Major Trauma Centre (MTC) located in Cambridge—the region with the highest rates of cycling in the UK[10]. In this region, 56.9% of people cycle at least once a week, compared to 38.8% in the second highest region [10]. Approximately one third of the population of Cambridge also cycle to work [10]. This high prevalence of cycling in the region of our MTC, allows us to provide a unique and valuable perspective on injuries sustained by cyclists.

## Materials and methods

This is a retrospective analysis of orthopaedic fractures sustained by all patients admitted to our MTC, due to injuries sustained whilst riding a bicycle, between 1st January 2012 and 31st December 2020. Patients involved in cycling-related accidents were identified using the Trauma Audit and Research Network database (TARN). Patients meeting all three of the TARN inclusion criteria are listed on this database: (1) Trauma patients regardless of age; (2) patients with a hospital stay of three or more nights, who are admitted to critical care or who die during hospital stay; (3) those whose isolated injuries fall into several categories, detailed in the appendix. This database contained information including date of injury, patient age, sex, injury mechanism, Glasgow Coma Scale score (GCS) at the scene, and Injury Severity Score (ISS). Using an electronic patient record system, further information was collected, including fracture locations, total number of fractures (accounting for multiple fractures to the same bone), and details of treatment received. Patients were divided into two groups according to injury mechanism: collision with motorised vehicle (including car, van, heavy goods vehicle, and motorcycle) and collision with non-motorised vehicles/objects (including collisions with other pedal cyclists, falls from a cycle, collisions with lampposts and signs, etc.). Comparisons of injury patterns/characteristics were made between these groups and according to patient age. The specific patient age categories shown were chosen to align with previous work characterising fractures sustained by cyclists [7].

All statistical analysis was performed using IBM SPSS Statistics 25. Chi-square tests were used to compare categorical variables.

## Results

A total of 737 patients were admitted to our MTC due to injuries sustained as a result of accidents involving patients on bicycles between January 2012 and December 2020. Of these patients, 292 (median age 50.5 years, interquartile range 38–60) sustained one or more fractures of the appendicular skeleton, with 225 (77.1%) being male and 67 being female (22.9%).

A total of 510 fractures to the lower limb, upper limb or pelvis (including acetabulum) were sustained, with 142 patients (48.6%) sustaining 2 or more fractures. Upper limb fractures were the most frequently observed (41.4% of total, *n* = 211), followed by lower limb fractures (33.9% of total, *n* = 173) and pelvic fractures (24.7% of total, *n* = 126). Fractures were most frequently observed in people in the > 50 years category, followed by the 19–49 years category (Table 1, *p* < 0.00001). This pattern remained when analysing male and female patients separately.

**Table 1:** Number of people within each age group who presented with upper limb, lower limb or pelvic fractures. (Individuals with fractures in more than one region have been included twice)

	< 12 years	13–18 years	19–50 years	> 50 years	Total
Upper limb	2	10	60	70	142
Lower limb	0	9	60	54	123
Pelvis	0	7	36	55	98
Total	2	26	156	179	363

### Fracture pattern

The clavicle was the most fractured upper limb bone (29.9% of upper limb fractures *n* = 63), whereas the most frequently fractured lower limb bone was the neck of femur (NOF) (25.4% of lower limb fractures *n* = 44). The breakdown of upper and lower limb fractures is shown in Table 2.

**Table 2:** Number of fractures in the lower and upper limb separated by the fracture location

Lower limb		Upper limb	
Fracture location	Number of fractures (%)	Fracture location	Number of fractures
Neck of femur	44 (25.4%)	Scapula	34 (16.1%)
Other femur	31 (17.9%)	Clavicle	63 (29.9%)
Patella	6 (3.5%)	Humerus	23 (10.9%)
Tibia	36 (20.8%)	Radius	43 (20.4%)
Fibula	27 (15.6%)	Ulna	21 (10.0%)
Ankle	10 (5.8%)	Hand	27 (12.8%)
Foot	19 (11.0%)	–	–
Total	173	Total	211

No significant difference was found in the prevalence of upper limb, lower limb, or pelvic fractures across different age groups. In males, consistent with the overall results, the clavicle was the most fractured upper limb bone (32.3% *n* = 52), whereas, in females, the radius was the most commonly fractured upper limb bone (24% *n* = 12). In the lower limb, NOF was the most fractured bone in males (28.4% *n* = 38), and the tibia was the most fractured bone in females (25.6% *n* = 10).

### Mechanism of injury

More patients sustained non-motorised fractures compared to motorised fractures (61.3% *n* = 179 compared to 38.7% *n* = 113). For motorised accidents, the highest prevalence was seen in the 19–49 years age group (52.2%, *n* = 59, *p* = 0.006), whereas non-motorised accidents were most prevalent in the > 50 years age group (56.4% *n* = 101 *p* = 0.006). The most frequently fractured lower limb bone during non-motorised accidents was the NOF (47.6% *n* = 39), whereas the tibia was the most fractured lower limb bone in motorised accidents (26.4% *n* = 24). (Table 3) In the upper limb, the clavicle was the most fractured bone in both motorised and non-motorised groups (26% *n* = 26 and 33.3% *n* = 37, respectively). In the lower limb, NOF (*p* < 0.00001) fractures were more common in non-motorised compared to motorised accidents, whereas fibula fractures were more likely to occur in motorised injuries (*p* = 0.04 (Table 3)).

On the other hand, in the upper limb, only scapula (*p* = 0.01) fractures were more likely to occur in motorised accidents. In terms of injury severity, the occurrence of an injury severity score (ISS) ≥ 16 was much more likely in the motorised compared to the non-motorised group (62.3% compared to 30.7% *p* = 0.0001).

### Management

Of the 292 patients who sustained at least 1 fracture, 176 (58.5%) required surgery. Of these, 133 (76%) were male, and 42 (23%) were female. Lower limb fractures more commonly required surgical treatment compared to upper limb fractures, such that 67.1% (*n* = 116) of lower limb fractures underwent surgery whereas only 34.6% (73) upper limb fractures required surgery.

Pelvic fractures were the most likely to undergo conservative management, with 24.6% (*n* = 31) managed surgically (*p* < 0.00001). The most likely lower limb fracture to be operated upon was NOF fractures, with 95.5% receiving surgery (*n* = 42 *p* = 0.00003), followed by tibia fractures where 81.1% were treated surgically (*n* = 31 *p* = 0.007). In upper limb fractures, the ulna was most likely to be operated upon (90.5% *n* = 19 *p* < 0.00001), followed by the humerus (56.5% *n* = 13 *p* = 0.02). The most likely age group to undergo surgery was the 13–18 years group, where 62.5% (*n* = 25) of fractures sustained in this age group received surgery (*p* = 0.01). Furthermore, for both upper (*p* = 0.0007) and lower limb (*p* = 0.005) fractures, individuals who had sustained a motorised injury were more likely to receive surgical management. Of the surgical procedures performed, the most common was open reduction and internal fixation (83.2% *n* = 183), followed by closed reduction and internal fixation (8.2% *n* = 18).

## Discussion

This study included data from 737 patients admitted for cycling-related injuries to our MTC between January 2012 and December 2020. From this sample, 39.6% of patients suffered an orthopaedic fracture (upper limb, lower limb, and pelvis), and of those, 48.6% sustained more than one fracture. The proportion of patients sustaining an orthopaedic fracture in this study was lower than the equivalent figure of 63.3% reported in by Tenenbaum et al. [7]. However, it is important to note that their study reported fracture characteristics specifically in e-cycle users, and so this difference may reflect an increased fracture risk in e-cycles when compared to all cyclists. Alternatively, this may be explained by a safety-in-numbers effect, whereby the risk of more high energy collisions is reduced in regions, such as ours, where cycling rates are high [11–13].

### Demographics

The largest proportion of fractures occurred in patients aged over 50; 49.3% of fractures. This finding may be related to increased bone resorption and density loss, with previous studies reporting a general increase in adult fracture risk with increasing age [14, 15]. Court-Brown et al. also report that 46.2% of almost 6000 fractures occurred in patients over 50, and so it is not surprising that our study shows cycling-related fractures were also most prevalent in this group [16]. However, it is important to consider other demographic factors when assessing cyclists’ fracture risk. It has previously been reported that male cyclists behave in a riskier manner than their female counterparts and are thus more likely to be involved in collisions [17, 18]. This may explain why over three-quarters of patients in our study were male, a finding reported by studies previously [7, 19–21]. Studies have also found that younger cyclists are at a higher risk of being involved in collisions [18, 22–24]. Previous studies suggest that this may be due to younger cyclists’ risk perception, increased risk taking behaviours and poorer knowledge of road traffic rules compared to older cyclists [23, 24]. While this was not directly reflected in our fracture distribution results, it is a factor that policymakers and healthcare providers should consider when designing safety interventions. For example, targeting education programmes and safety products to these demographics could help cyclists and drivers be less complacent and may, in turn, reduce collisions and hospital admissions. Furthermore, given that our centre’s population has a significant number of new cyclists each year in the form of university students due to the presence of two universities with a combined student population of approximately 62,000, these programmes may be targeted to those who may be inexperienced in road cycling and therefore at increased risk of a collision [25].

With respect to the general adult population, studies have shown several modifiable factors may be responsible for causing a cycling accident. These include road maintenance factors such as potholes or wet/oily surfaces, road infrastructure such as a reduction in road speed limit, cycle only lanes and driver/cyclist education [12, 26–29]. Targeting these factors in safety interventions may help to reduce the risk of cycling accidents.

### Fracture pattern

Our results, demonstrating a greater number of upper limb compared to lower limb fractures, disagree with similar research [7]. Previous work has suggested patient age may affect the likelihood of fracturing a specific bone; for example, Tenenbaum et al. report that pelvic fractures were more common in elderly age groups [7]. Our study does not replicate this, with no significant association seen between age and risk of fracturing a specific bone. While skull and facial bone fractures were not included in our analysis, we noted that they were disproportionately common in the 19–49 age group (70.2% of these fractures). This may be partly explained by the fact that helmet use has been shown to protect against these fractures and that these patients are less likely to use one [30, 31]. Despite this, and several safety campaigns in schools and commercially, there is an issue with the compliance of helmet use, one study finding 30% of those aged 25–44 never use a helmet [19]. Therefore, increasing helmet uptake may be another important aspect of injury prevention.

### Injury mechanism

When analysing patients according to the mechanism of injury, we found that most collisions did not involve a motor vehicle, which is replicated in other reports [13, 20, 21, 32]. This may, however, not be the case in every UK region, as our city may have more cycling-related infrastructure helping reduce the chances of a motorised accident. Drivers may also be hypervigilant of cyclists in a city where cycling is more common, affecting road habits, making it safer for both parties [11, 33]. It is important to evaluate the mechanism of injury as this may affect fracture distribution and injury severity. For instance, in lower limb fractures, a NOF fracture was more likely to occur in a non-motor accident, whilst a fibular fracture was more likely in motor accidents. This could be due to non-motorised accidents representing a fall onto the hip, whereas motor vehicle accidents may lead to the vehicle bumper hitting the fibula directly. Furthermore, the risk of major trauma, defined as ISS > 16, was greater following motorised accidents. It may thus be said that although non-motorised accidents are more common, motorised collisions are more severe. It is therefore important to target safety interventions at both cyclists and motorised vehicle drivers.

### Fracture management

A total of 176 of the 292 (58.5%) patients sustaining an orthopaedic fracture required surgical treatment. In both upper limb and lower limb fractures, surgical management was more likely following motorised than non-motorised accidents. This association is replicated in previous work and emphasizes the severity of motorised accidents [7]. Surgical management was also more likely in patients aged 13–18. Patients sustaining a lower limb fracture, specifically NOF and tibia, were more likely to require surgical intervention, whilst in the upper limb, the ulna and humerus were more likely to require surgery. These factors have implications for practitioners in terms of risk assessment and clinical decision-making following the admission of a cycling injury patient.

The data demonstrated a high prevalence of fractures amongst patients admitted due to cycling-related accidents, and the burden managing these may have on a trauma service. Research shows that cyclists involved in a previous “near miss” have a heightened perception of traffic risk [34, 35]. This suggests that those with an awareness of the consequences of dangerous cycling are less likely to engage in such behaviours. Although there is currently no supporting evidence, one way this awareness could be increased is through trauma surgeons themselves visiting schools, universities, and workplaces to educate about the importance of cycling safety. Similar schemes have been used in schools by other emergency services such as the police and fire service to reduce crime and increase fire safety.

### Limitations

It must be acknowledged that this study is not without limitations. Firstly, only patients who were admitted into the hospital were included in the analysis, whilst patients discharged following treatment in the emergency department were not. It may therefore be the case that this study provides an overestimate of fracture prevalence and severity following cycling accidents. Furthermore, our database did not record orthopaedic injuries other than fractures, and so we were unable to analyse non-fracture orthopaedic injuries.

This study specifically focusses on orthopaedic fractures which are likely to be managed by orthopaedic surgeons across most hospitals. Therefore, whilst skull, facial and spinal fractures are commonly seen following cycling accidents, they were not included in our analysis. Such fractures are often referred to and treated by neurosurgeons or maxillofacial surgeons and are associated with brain and spinal cord injuries which do not form a part of the workload of an orthopaedic surgeon (Table 3).

**Table 3:** Number of fractures in each bone, according to the mechanism of injury

Lower limb			Upper limb		
Fracture location	Motorised	Non-motorised	Fracture location	Motorised	Non-motorised
Neck of femur	5	39	Scapula	23	11
Other femur	19	12	Clavicle	26	37
Patella	4	2	Humerus	11	12
Tibia	24	12	Radius	17	26
Fibula	19	8	Ulna	6	15
Foot	12	7	Hand	17	10
Ankle	8	2	–	–	–
Total	91	82	Total	100	111

## Conclusion

This study adds to the current paucity of literature characterising fractures sustained as a result of cycling-related injuries. Such research is becoming increasingly important in light of recent cycling promotion schemes. Our results show approximately 40% of patients admitted due to cycling-related injuries sustained a fracture, with upper limb fractures being more common. Fractures were more common in those aged over 50. Patients who sustained a motorised injury were more likely to require surgical management. The unique data presented are of importance in informing clinicians about injury patterns seen following cycling accidents and in aiding policymakers to design suitable safety interventions.

## Supplementary Information

Below is the link to the electronic supplementary material.

Supplementary file1 (DOCX 19 kb)

## Data Availability

Not applicable.
