# Supplementary material for: Cycling-related orthopaedic fractures admitted to the Major Trauma Centre in the cycling capital of the UK
Source: Arch Orthop Trauma Surg. 2021 Aug 3;142(10):2747–53. doi: 10.1007/s00402-021-04097-3 (PMC8330467; doi:10.1007/s00402-021-04097-3)
Supplement: Supplementary file 1 — Supplementary file1 (DOCX 19 kb) [file 402_2021_4097_MOESM1_ESM.docx]

**TARN injury criteria**

Patients who meet the two other TARN inclusion criteria and with at least one of the following injuries are eligible for inclusion in the TARN database. Visit <https://www.nbt.nhs.uk/sites/default/files/attachments/TARN%20manual%20-%20February%202014.pdf> for further information.

**Head injuries:**

All brain or skull injuries such as

- Skull fracture
- Traumatic haemorrhage
- Brain contusion
- Brain laceration/penetration
- Traumatic brain swelling
- Vascular injury
- Nerve injury
- Diffuse axonal injury

**Facial injuries:**

- Unstable, comminuted, open or compound facial fracture. Fracture with significant displacement
- Orbital blow out fracture
- Le fort fracture
- Pan-facial fracture
- Injury to branches of external carotid artery
- Optic/facial nerve injury
- Eye avulsion
- Traumatic retinal detachment
- Globe rupture

**Neck injuries:**

- Injury to major vessels: carotid and vertebral artery, jugular vein
- Organ injury
- Hyoid fracture

**Thoracic injuries:**

All internal injuries such as

- Vagus nerve injury
- Vascular injury
- Organ injury
- Traumatic sternum fracture
- Traumatic rib fracture
- Flail chest
- Haemothorax
- Pneumothorax
- Haemomediastinum
- Pneumomediastinum

**Abdominal injuries:**

All internal injuries such as

- Vagus nerve injury
- Vascular injury
- Organ injury e.g. contusion, laceration, transection, avulsion, perforation, rupture (including genitourinary organs)
- Retroperitoneal haemorrhage

**Spinal injuries:**

- Traumatic vertebral fracture
- Vertebral dislocation
- Disk injury
- Nerve root injury
- Brachial plexus injury
- Cord injury

**Femoral injuries:**

- Hip fractures (neck of femur, intertrochanteric, pertrochanteric/greater trochanter, basi-cervical, intracapsular, subcapital) in patients aged < 65 years
- Head, shaft, distal or subtrochanteric fracture in patients of any age
- Femoral vessel injury
- Femoral nerve injury

**Pelvis/acetabulum:**

- Single pubic rami fracture in patients aged <65
- Patients of all ages with multiple pubic rami fractures
- Ischium, sacrum, coccyx or ileum fracture
- Acetabulum fracture
- Symphysis pubis joint injury
- Sacro-iliac joint injury
- Lateral compression fracture
- Anterior posterior compression fracture
- Open book fracture
- Vertical sheer fracture
- Malgaigne fracture

**Upper limb or lower leg injuries:**

Upper limb refers to scapula, clavicle, humerus, radius and ulna

Lower leg refers to patella, tibia, fibula, calcaneus, talus – femoral injuries are described separately above

- Open (compound) fractures and/or dislocations
- Total crush injury
- Traumatic amputation
- Fractures and/or dislocations of multiple limbs
- Transected vessels
- Sciatic nerve injury

**Nerve injuries:**

- Any injury to sciatic, facial, femoral or cranial nerve

**Vessel injuries:**

- All injuries to femoral, neck, facial, cranial, thoracic or abdominal vessels. Transection or major disruption of any other vessel

**Hand and feet injuries:**

- Crush of entire hand or foot
- Amputation of hand or foot

**Burn or inhalation injuries:**

- Full thickness burns
- >10% total body surface area burn
- Inhalation injury

**Other injuries:**

- Electrocution injuries
- Full thickness frostbite
- Asphyxia (with loss of consciousness)
- Drowning (with loss of consciousness)
- Skin lacerations with blood loss >20%
- Penetrating injuries to skin with blood loss >20%
- Degloving injury
